# Gastroparesis: Concepts, Controversies, and Challenges

**DOI:** 10.6064/2012/424802

**Published:** 2012-08-08

**Authors:** Klaus Bielefeldt

**Affiliations:** Division of Gastroenterology, University of Pittsburgh Medical Center, 200 Lothrop Street, Pittsburgh, PA 15213, USA

## Abstract

Patients with gastroparesis often present a challenge to the treating physician. Postprandial symptoms with nausea and vomiting may not only lead to nutritional and metabolic consequences, but also cause significant disruptions to social activities that often center around food. While the definition of gastroparesis focuses on impaired gastric emptying, treatment options that affect gastric function are limited and often disappointing. The female predominance, the mostly idiopathic nature of the illness with a common history of abuse, and coexisting anxiety or depression show parallels with other functional disorders of the gastrointestinal tract. These parallels provided the rationale for some initial studies investigating alternative therapies that target the brain rather than the stomach. This emerging shift in medical therapy comes at a time when clinical studies suggest that gastric electrical stimulation may exert its effects by modulating visceral sensory processing rather than altering gastric motility. Physiologic and detailed anatomic investigations also support a more complex picture with different disease mechanisms, ranging from impaired accommodation to apparent visceral hypersensitivity or decreased interstitial cells of Cajal to inflammatory infiltration of myenteric ganglia. Delayed gastric emptying remains the endophenotype defining gastroparesis. However, our treatment options go beyond prokinetics and may allow us to improve the quality of life of affected individuals.

## 1. Introduction

Recent reports suggest that within the last two decades, gastroparesis may have developed from a rare disorder to an increasingly common and often frustrating problem with at times prolonged hospitalizations [1]. Extrapolating from symptoms on the presence of impaired gastric function, Rey and colleagues even speculated about gastroparesis affecting up to 2% of the population, likening the clinically identified patients to the tip of an iceberg [2]. The disease has morphed from its initial description as complication of long-standing diabetes or prior gastric surgery into a mostly idiopathic disorder that primarily affects women [3–8]. While the phenotypic definition of the illness is based on dysmotility, treatments with a focus on accelerating the delayed gastric emptying still leave patients and physicians disappointed. This limited efficacy of available therapies keeps many patients struggling when it comes to meeting their daily caloric needs [9]. Not being able to eat without experiencing symptoms impacts more than a patient's energy balance. Many of our social interactions revolve around food or drinks, adding to the indirect burden of this disease [10]. The clinical presentation with discomfort and vomiting, emerging nutritional problems, the few available prokinetics as the first choice of therapy in a disease defined by altered motility and their limited efficacy often lead to frustration of patients and physicians. Thus, gastroparesis is a difficult problem, often making patients into “difficult” patients. 

Looking at this disease and its challenges, I want to address key questions that patients typically raise and that we need to understand as physicians or investigators dealing with this problem. What is gastroparesis? How can we confirm the presence of gastroparesis? How common is this disease? What causes gastroparesis? What is the prognosis? What are the mechanisms of impaired gastric function? What treatment options do we have? While many of these questions cannot be fully answered, we have gained significant insight into the epidemiology and pathogenesis of gastroparesis, insight that may affect our diagnostic and/or therapeutic approaches. Reviewing the available information also points into potential directions for future studies. All of these sections will therefore include controversial or emerging ideas that may shape our views on or management of gastroparesis in the years to come.

## 2. Definition of Gastroparesis

Relying on published consensus statements, gastroparesis is defined by the presence of dyspeptic symptoms and the documented delay in gastric emptying of ingested nutrients in the absence of gastric outlet obstruction. A third factor, duration of symptoms, is generally added, as many acute illnesses or abdominal operations transiently impair stomach function, but typically resolve within a relatively short-time period [11, 12].

Traditionally, gastroparesis was thought to be characterized by anorexia and postprandial symptoms with nausea, vomiting, bloating, early satiation and fullness. Pain was not considered to be typical and raised the question of functional dyspepsia, more recently classified as postprandial distress syndrome [12, 13]. However, several case series recently highlighted not just the potential presence of pain in patients with gastroparesis, but also its importance with 20–40% of the affected individuals rating it as their dominant symptom [10, 14–17]. The overlap between gastroparesis and functional dyspepsia extends beyond pain. Using standardized questionnaires, several large case series described symptom severity scores in patients with functional dyspepsia and gastroparesis, not showing a significant difference between the two patient groups (Table 1). Consistent with this scenario, nearly 90% of a large and well-characterized patient group with idiopathic gastroparesis met diagnostic criteria for functional dyspepsia [14]. Conversely, about one-third of patients with functional dyspepsia have delayed gastric emptying [18]. Thus, the traditional boundaries between functional dyspepsia and gastroparesis have become blurry with the presence or absence of delayed emptying being the primary difference.

Even in the absence of subjective symptoms, delayed gastric emptying may lead to nutritional and metabolic consequences. Detailed data from a national research consortium demonstrate the impact of the often limited ability of patients to tolerate oral intake; the overall caloric intake was deficient and lacked several essential micronutrients [9]. Changes in gastric emptying may alter postprandial glucose concentrations and thereby significantly affect diabetic control [19, 20]. Insulin treatment is more difficult with erratic gastric emptying and often requires adjustments in diabetics with gastroparesis [21]. If occurring in isolation (i.e., without other symptoms), these scenarios do not meet the accepted consensus criteria. However, the detrimental nutritional and metabolic consequences may be sufficient to consider the diagnosis of gastroparesis.

Postprandial discomfort or fullness and early satiation not only characterize gastroparesis and functional dyspepsia, but also are the main mechanism of restrictive bariatric surgeries. With the obesity epidemic in most developed countries, could a reversible induction of gastroparesis be beneficial? Exenatide and other GLP1 receptor agonists indeed trigger a delay in gastric emptying, which may be beneficial for type II diabetics due to the blunted postprandial hyperglycemia [22]. The associated anorexia contributes to weight loss, another beneficial effect in persons with metabolic syndrome. Dose-limiting symptoms reminiscent of gastroparesis are seen initially in more than 25% but decrease over time and lead to discontinuation in about 2–5% [23, 24]; however, the potential of pancreatitis has limited the more widespread use of these agents in the management of obesity [25]. Despite this caveat, the data suggest that intentional and reversible delay in gastric emptying could provide an alternative management option in obese patients(see Box 1).

## 3. Clinical Testing

If the delay in gastric emptying indeed is indeed the key difference between gastroparesis and other functional disorders affecting the upper gastrointestinal tract, then appropriate assessment of gastricfunction constitutes the endophenotype and establishes the disease. A variety of tests have been developed to measure gastric emptying. Since its initial description more than 40 years ago, scintigraphic assessment has been widely accepted and has become the “gold standard” for the diagnosis of gastroparesis [32]. Early on, investigators recognized the importance of volume, caloric content, nutrient composition, and consistency in determining the rate of gastric emptying [33–35]. To minimize variability and allow comparisons, the test should use a predefined volume and caloric load and should be carried out over 4 h [32, 36]. Using such approaches, repeat testing shows an acceptable intraindividual reproducibility despite significant variability between persons [37]. 

**Figure 1:**
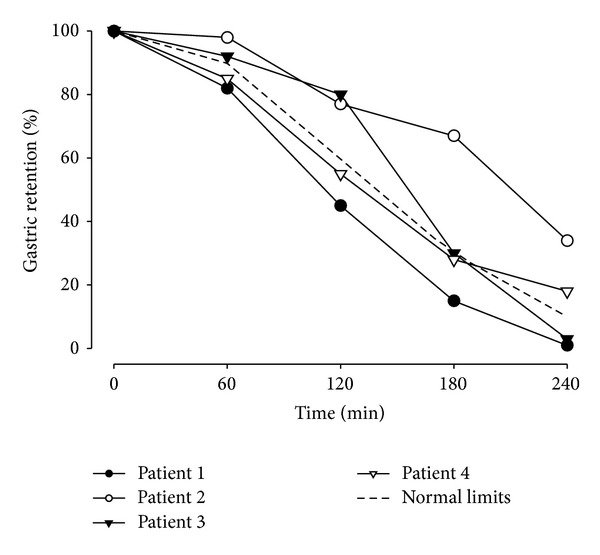
Sample emptying curves showing the results of scintigraphic studies of gastric emptying for four different patients as compared to the reference of control persons indicated by the dotted line. While the findings for patients 1 and 2 are unequivocal with normal (1) and slow (2) emptying, the other two patients have less consistent results with delay at early (3) or late (4) times points only.

Figure 1 shows the results of four gastric emptying tests obtained in patients with dyspeptic symptoms. The sample curves represent four distinctly different findings. Two of the patients had consistently normal or abnormal rates of gastric retention, allowing a clear conclusion. However, the other two showed inconsistent results over time with delayed emptying at one time point only. Such scenarios are not uncommon. A large study indicates that about 1 in 6 patients with delayed emptying at 2 hours will have normal emptying at 4 hours; more importantly, nearly half of the patients with abnormal results at 4 h had normal findings at 2 hours [38]. Consistent with these results, the percentage of patients with dyspeptic symptoms diagnosed with gastroparesis may increase from about 33% to close to 60% if we focus on the longer test duration [39]. The picture gets even murkier if we look at gastric emptying for both solids and liquids. Discrepancies have been seen in about one-third of the patients with one in four patients with normal emptying of solids having delayed emptying of liquids [40, 41]. As gastric emptying studies are considered the “gold standard” in the diagnosis of gastroparesis, we cannot validate the test against other standards. However, we can assess the correlation between symptoms and various endpoints of a gastric emptying study to help decide on appropriate diagnostic criteria. A recent study demonstrated a limited correlation between symptom severity and gastric emptying with gastric retention of solids at 4 hours explaining less than 15% of the variance in symptom severity ratings [41]. These results are consistent with more detailed physiologic testing in patients with gastroparesis, which suggests that mechanisms other than emptying, such as impaired accommodation and hypersensitivity, contribute to early satiation, weight loss, and pain [42].

Despite these shortcomings, impaired gastric emptying remains the defining endophenotype of gastroparesis. Thus, other tests assessing gastric emptying have been developed. Ultrasound can be used to monitor antral contractions and determine the volume changes after a mixed meal. The results correlate reasonably well with scintigraphic determination of gastric emptying [43]. However, interference due to intraluminal air and the increasing identification of gastroparesis in obese patients limits the clinical utility of this approach [14]. Magnetic resonance imaging can similarly address contractions and volume changes, thus allowing to determine accommodation and emptying after a test meal [44]. Cost and complexity of data analysis argue against a widespread use of this approach. Using stable nonradioactive carbon isotopes (e.g., labeled fatty acids) in a test meal, one can determine time-dependent changes in these isotopes in the exhaled air, as the ingested material is absorbed and metabolized once it exited the stomach. Such breath tests have an acceptable intra-individual variability of around 15% and correlate reasonably well with scintigraphic assessments of gastric emptying [45, 46]. More recently a wireless capsule was introduced as an alternative technique for gastric emptying. Being a large particle, it will typically not exit the stomach with the meal, but will be expelled during the repeated high amplitude contractions of the phase III activity of the migrating motor complex, which resumes after completion of gastric emptying. The entry into the duodenum is indicated by the sudden jump in pH and correlates reasonably well with scintigraphic findings [47]. Recordings of intraluminal pressure or gastric electrical activity have been proposed as diagnostic tests in patients with dyspeptic symptoms. While they often show abnormalities in patients with gastroparesis, they can at best correlate with but do not assess transit. As prolonged retention of ingested material defines the currently accepted endophenotype, assessment of contractile or electrical activity may provide additional mechanistic insights, but dose not truly aide in the diagnosis of gastroparesis. 

In addition to considerations about testing strategies and potential targets for their treatment, clinicians need to pay attention to confounders as results are affected by medications and metabolic parameters. Most physicians are aware of opioid effects on gastrointestinal motility, which should be kept in mind because about 20–40% of patients with gastroparesis use such agents at least intermittently [6, 10, 48–50]. Many antiemetics, antidepressants, and other commonly used agents have anticholinergic effects and may thus interfere with gastric emptying [51]. Gastroparesis can obviously be a complication of long-standing diabetes; it is thus important to consider the impact of hyperglycemia, which reversibly impairs contractility and emptying [19, 52], with improvement of gastric emptying and symptoms after appropriate treatment [53](see Box 2).

## 4. Epidemiology and Prognosis

**Figure 2:**
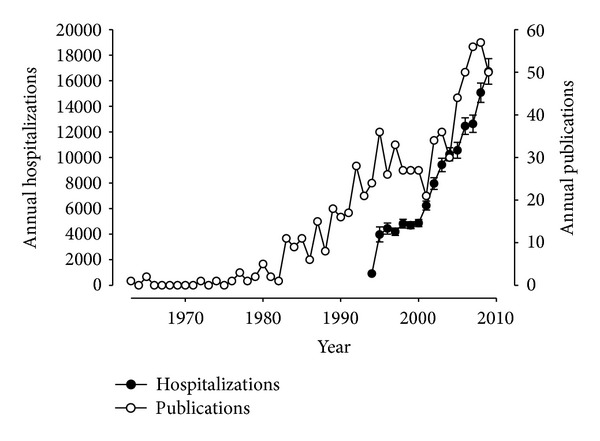
Time trends in abstracted English-language publications about gastroparesis retrieved from the PubMed data bank (white circles) and reported annual hospitalizations (black circles) for gastroparesis as primary diagnosis (based on data from the Nationwide Inpatient Sample of the Agency for Healthcare Research and Quality).

Only one single study has assessed the prevalence of gastroparesis. Based on clinical records, about 30 per 100,000 persons will have sought medical attention for gastroparesis with an increasing prevalence with age [54]. During 1996 to 2006, there was no change in the incidence or prevalence of gastroparesis. These epidemiologic data stand in stark contrast with increasing hospitalization rates for gastroparesis [1]. Interestingly, the rise by more than 180% is even more striking if we extend the time period with an 18-fold increase in inpatient treatments for gastroparesis as the primary diagnosis between 1994 and 2009. As shown in Figure 2, this increase is preceded by an increase in annual publications focusing on gastroparesis. As this time period saw the above-mentioned rise in hospitalizations due to gastroparesis, recognition or different diagnostic labeling is indeed the likely cause for this discrepancy.

**Figure 3:**
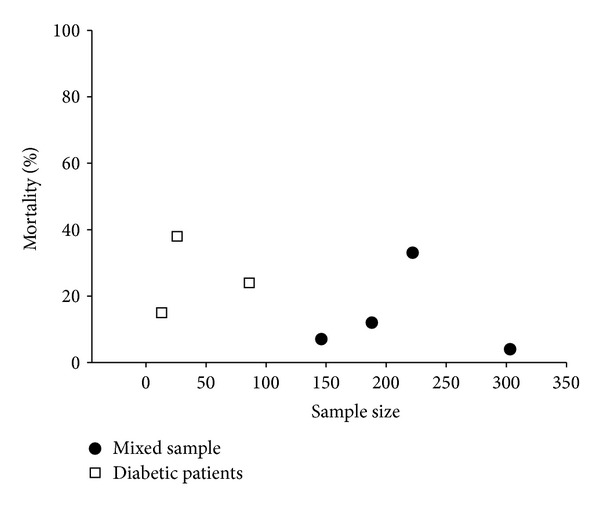
Reported mortality in published case series of patients with gastroparesis (black circles: mixed etiology; white squares: diabetic gastroparesis).

Unfortunately, relatively little information is available to address the natural course of gastroparesis. As shown in Figure 3, case series report mortality rates between 4% and 38% with the best outcomes described for a largely outpatient-based group of patients followed for about 2 years and the highest death rate for diabetic patients with gastroparesis requiring nutritional support [6, 15, 54–58]. Only one population-based study compared the observed and expected mortality of patients with gastroparesis, demonstrating an significantly higher death rate in patients, which was largely due to cardiovascular comorbidity in diabetic patients [54]. The duration of diabetes and the existence of secondary complications, but not the presence of dyspeptic symptoms and/or documented delay in gastric, emptying were poor outcome predictors in diabetic patients followed for close to 10 years [59, 60]. Consistent with these data, no death was reported when gastroparesis was the primary diagnosis rather than a complication of diabetes in hospitalized patients [61].

Even if gastroparesis is not associated with increased mortality, it nonetheless carries a significant burden with an increase in hospitalizations [60, 62] and a decrease in quality of life [7, 10, 26]. A retrospective study suggests that about one third of patients with gastroparesis will require hospitalizations for symptom exacerbations or nutritional support throughout a year [6]. Inpatient treatment is more commonly required in diabetics than patients with idiopathic forms of gastroparesis [8]. These data are consistent with results from the only population-based investigation, showing the need for admission and therapeutic interventions in one quarter of the patients during a follow-up period of five years [54]. A relatively small number of patients requires more frequent or prolonged hospitalizations, often triggered by poor diabetic control, infections, medication intolerances, and/or poor adherence to treatment recommendations [6, 63]. Interestingly, emotional problems strongly correlate with the overall symptom severity and also confound the utilization of healthcare resources [7, 8, 10].

Beyond data on mortality and information about hospitalizations, we have limited insight into the natural course of gastroparesis. A small number of diabetic patients followed over more than 10 years had stable symptoms and gastric emptying rates, arguing against rapid and/or common progression in this group [56]. More than 70% of patients seen in a tertiary referral center required ongoing medical therapy during a time period of about 10 years, indicating that at least some symptoms indeed persist in the majority of affected individuals [15]. Several case series indicate that postinfectious forms of gastroparesis may be an exception from this rule with symptom resolution or at least improvement occurring in many patients within one year of the inciting event [14, 64–66]. 

The chronic illness certainly takes a toll with significant impairment of quality of life with significant lower scores on measures assessing physical and mental/emotional function [8, 10, 26]. At least one study pointed at an indirect impact of the chronic illness with high rates of un- or underemployment and, perhaps as a result, a high number of patients with low household incomes [10]. While a more recently published study of nearly 400 patients showed that patients reported median household incomes close to the national average, it also demonstrated the economic burden with less than half of the patients being employed at the time of enrollment and nearly one third having high rates of work absenteeism due to their disease [8] (see Box 3).

## 5. Etiology

A great many diseases can cause impairment of gastric function; the growing list ranges from functional changes after surgery to inherited disorders, neurologic diseases, connective tissue disorders, metabolic abnormalities, most importantly diabetes, and infections [64, 67–72]. As shown in Table 2, the vast majority of patients fall into three groups: patients with long-standing diabetes, patients with prior foregut surgery, and patients without identifiable cause (idiopathic gastroparesis). Except for studies with a focus on hospitalized patients and patients undergoing implantation of a gastric electrical stimulator, essentially all recent case series show that no identifiable cause for gastroparesis can be found in 50–70% of patients.

More than 20 years ago, researchers described a subgroup of patients with idiopathic gastroparesis who could recall an acute infection preceding the onset of their more chronic symptoms [78]. Similar scenarios have since been reported by others with postinfectious gastroparesis accounting for up to 20% of the idiopathic forms of the illness [14, 64–66]. While the exact mechanisms have not been identified, the temporal sequence certainly suggests causality and points at a potential inflammatory or immune-mediated process with secondary changes of nerves, interstitial cells of Cajal and/or muscle cells as the key structural elements involved in normal gastric motor function. Considering the potential role of infection, several investigators have addressed the possible contribution of mucosal inflammation. A small study indicated a higher likelihood of impaired gastric emptying in patients with chronic *Helicobacter pylori* infection [79]. However, the degree of microscopic inflammation did not correlate with emptying delay in children with dyspeptic symptoms [80, 81]. When including control groups with dyspeptic symptoms but without infection, others did not confirm a relationship between the presence of chronic infectious gastritis and a delay in gastric emptying [82–85]. 

Epidemiologic data also identified some contributing factors. Increasing age and female gender both correlate with a higher risk of impaired gastric function [36, 54, 86–88]. Consistent with the potential importance of gender, most studies of gastroparesis show a very striking female predominance with about 70–80% (Table 2). Interestingly, about two-thirds of the women with idiopathic gastroparesis report prior physical or sexual abuse [15]. Two recent studies demonstrated that anxiety and depression ratings correlate with the perceived severity of gastroparesis symptoms [7, 10]. The female predominance, the history of prior abuse, and the importance of emotional factors all show parallels to many other functional disorders, where central mechanisms significantly contribute to disease development or symptom severity [89–92] (see Box 4).

## 6. Disease Mechanisms 

With altered motility defining the endophenotype of gastroparesis, many investigators looked for changes in the different cellular structures that control normal motor function of the stomach. As vagotomy impairs gastric emptying and as long-standing diabetes is associated with gastroparesis and neuropathy, changes in vagal innervation could indeed contribute to the development of gastroparesis. Diabetic patients with gastroparesis show evidence of autonomic neuropathy with lower vagal tone [93]. Studies in humans did not show differences in the microscopic appearance of the subdiaphragmatic vagus in diabetic patients [94]. However, the postprandial increase in PPY and ghrelin is blunted or absent in patients with diabetic or postsurgical gastroparesis, but not idiopathic gastroparesis, suggesting that the function rather than the light-microscopic structure of vagal pathways is abnormal in this subset of patients [95, 96]. Looking beyond just vagal pathways, several investigators described changes in nerve fiber density within the stomach or ultrastructural changes in nerve fibers of patients with gastroparesis [97–100]. While the aggregate data thus suggest a change in gastric innervation, the complex innervation of the stomach with extrinsic and intrinsic nerves does not enable us to determine origin and type of affected nerves fibers. Immunohistochemical stains have identified distinct subgroup of enteric neurons [101, 102]. Focusing on the cell bodies of the enteric nervous system, the total number has been reported to be similar [94] or lower [103, 104] in patients with gastroparesis. Relatively little information about the immunohistochemical phenotype of affected enteric neurons is available. Two independent investigations indicate a decrease in neurons expressing neuronal nitric oxide synthase with other markers not showing consistent differences between patients and controls [98, 105]. Beyond the potential impact of poor metabolic control in diabetic patients, two possible mechanisms may contribute to the changes in gastric innervation. A mild lymphocytic infiltration of enteric ganglia has been observed, which was more common in patients with diabetic compared to idiopathic forms of gastroparesis [104], while another group reported such changes as being more commonly associated with idiopathic gastroparesis [106]. Autoantibodies have been described which may target neuronal elements leading to impaired gastric function [107].

The interstitial cells of Cajal (ICC) have emerged as important determinant of normal gastric function [108]. A decrease in ICC numbers has been reported in patients with diabetic [98, 99, 104, 105, 109, 110] and idiopathic gastroparesis [98, 103, 104, 109–111], affecting perhaps one-third of the patients examined. Consistent with the role of ICC in gastric physiology, one group has observed a correlation between low ICC numbers and abnormalities in gastric electrical activity (tachygastria) [109, 110]. However, this correlation did not extend beyond changes in gastric electrical phenomena, as only the subgroup of diabetic patients showed severely delayed emptying in one study and as ICC loss did not predict symptom severity [106, 110]. ICC progenitor cells have been identified in the gastric wall [112]; it is unclear whether the decrease in ICC numbers is reversible and may in part be related to poor metabolic control or immune activation. Lastly, changes in the muscular layer with infiltration of eosinophils or macrophages and some degree of fibrosis have been reported in some cases [98, 100, 113, 114]. The relevance of these findings remains unclear (see Box 5).

## 7. Treatment

### 7.1. Dietary Therapy

Impairment of gastric function is associated with symptoms that are typically exacerbated by food intake, turning eating into a dreaded chore and interfering with many of the social activities that revolve around food intake [10]. In healthy individuals, gastric emptying is affected by nutrient density and food consistency [34, 115, 116]. Thus, dietary adjustments play a key role in limiting symptoms. In addition, nutritional therapy should prevent deficiencies that can be commonly found in patients with gastroparesis [9]. Despite the unquestioned importance of diet in the management of gastroparesis, no study has systematically addressed the impact of changes in food intake. In a small study, medical and dietary interventions were rated as most helpful by a similar number of patients [10]. General recommendations focus on volume and frequency of meals, stress the importance of low fat and fiber content, and suggest soft or even liquid nutrients for patients with more significant symptoms [11, 12, 117–119]. In diabetic patients, dietary management is even more important, as it obviously affects glucose levels and insulin therapy; moreover, The relationship between blood sugars, insulin therapy, and gastric emptying is more complex, as hyperglycemia impairs gastric emptying; conversely, improved diabetic control can also improve gastric function [53]. As patients often face nutritional deficiencies, they should take a multivitamin on a regular basis.

### 7.2. Accelerating Emptying

Considering the definition of gastroparesis by delayed gastric emptying, many patients receive prokinetic agents, with reported percentages ranging from 55% to 95% [6, 8, 10, 15]. After withdrawal of cisapride from the market, the most commonly prescribed agents are metoclopramide and erythromycin [6, 8, 117, 120]. 

Three randomized controlled trials compared the effects of metoclopramide with placebo with one additional study examining the acute effects on gastric emptying [121–125]. Within the timeframe of the trial (three weeks), overall symptoms improved by about 50%. Adverse effects were relatively common, affecting 25–50% of the patients and included drowsiness, psychomotor agitation, tremor and one dystonic reaction in these initial studies. With the more widespread use of metoclopramide, reports about side effects surfaced, focusing on the extrapyramidal motor disorders as the primary manifestation [126, 127]. Metoclopramide's mechanisms of action as a dopamine antagonist and the relatively high incidence of such motor effects even led to the use of its use as a provocative test in patients with suspected extrapyramidal motor disorders [128]. While most of these adverse effects are dose-dependent and reversible, concerns about the development of tardive dyskinesia, a typically irreversible and potentially devastating syndrome, surfaced [120, 129–132]. Some of the key risk factors for the development of tardive dyskinesia mirror are also risk factors for gastroparesis, namely, female gender, older age, and diabetes and have not necessarily affected prescribing patterns for metoclopramide [8, 132]. However, treatment duration was the one risk factor that can be targeted; expert consensus thus emphasizes the importance of patient education about the risk of metoclopramide therapy and the need to limit the cumulative daily dose and duration of its use [120, 133]. The related dopamine antagonist domperidone does not penetrate the blood brain barrier and has similar efficacy in small trials but a better side effect profile compared with metoclopramide [134, 135]. However, the agent has not been approved by the Federal Drug Administration in the United States and is only available with special approval by institutional review boards.

Soon after the description of motilin effects on gastric emptying investigators reported similar action after administration of erythromycin in humans [136, 137]. Acute infusion of erythromycin significantly accelerated gastric emptying in diabetic patients with gastroparesis [138]. This initial study already reported that use of an oral preparation for four weeks was still associated with improved emptying, but showed less of an effect. These findings were ultimately attributed to tachyphylaxis and led to the search for other agents without antibiotic effects and with slower development of receptor desensitization [139]. Three different motilin agonists have since been studied in large trials; despite their effects on gastric emptying, symptoms were not significantly improved compared to placebo [75, 140, 141]. Consistent with these results, short-term therapy with erythromycin effectively accelerated gastric emptying, but did not improve overall symptom severity [142]. A more detailed analysis of symptom clusters suggests an improvement of bloating, which is often quite severe and difficult to influence, thus still providing a limited rationale for its clinical use in gastroparesis [142, 143]. 

Recently, ghrelin has attracted significant interest as a possible prokinetic in patients with delayed gastric emptying. Patients with diabetic gastroparesis have low ghrelin levels [96]. When used acutely, ghrelin infusion accelerated gastric emptying in diabetic patients and patients with idiopathic forms of gastroparesis [144, 145]. A synthetic ghrelin agonist similarly improved emptying and had some beneficial effects on symptoms during short-term administration [146–148].

### 7.3. Targeting Accommodation

Abnormal gastric motility affects more than emptying, and several studies have demonstrated the importance of impaired accommodation, which may contribute to postprandial fullness, early satiation [42, 149]. Clonidine acutely decreased symptoms in patients with dyspeptic symptoms and diabetic gastroparesis [150, 151]. While this may be partly due to changes in accommodation, the effects are confounded by the central effects of the *α* agonist with decrease in visceral sensitivity [152]. Considering the hypotensive effects of clonidine, other investigators tested the effects of buspirone on gastric function and showed fundic relaxation with a slowing of gastric emptying in healthy volunteers [153]. Studies were limited to acute interventions and do not allow conclusions about the long-term effect of interventions targeting gastric accommodation. 

### 7.4. Targeting Pyloric Function

Conceptually, gastric retention could be due to impaired relaxation of sphincteric muscles within the pyloric channel, the mechanism of hypertrophic pyloric stenosis of infancy. This disease is caused by a lack of normal inhibitory innervation through nitric oxide-producing intrinsic neurons [154]. Interestingly, a lower density of neurons expressing nitric oxide synthase has been shown in patients with diabetic gastroparesis [98, 105]. The demonstration of increased contractile amplitudes within the pyloric channel of affected patients led to the hypothesis that impaired sphincter relaxation contributes to the development of gastroparesis. As nitric oxide exerts its effect through cyclic GMP as a mediator, delaying the breakdown of the second messenger should mimic effects of nitric oxide release. However, sildenafil did not improve emptying in a pilot study of patients with diabetic gastroparesis [155]. Based on a similar rationale, botulinum toxin was injected into the pyloric area during endoscopy. Initial reports were optimistic, reporting symptomatic improvement and acceleration of gastric emptying [50, 74, 156–159]. However, two controlled trials did not demonstrate superiority over placebo [76, 160] (see Box 6).

### 7.5. Targeting Symptoms

Nausea and vomiting are dominant symptoms of gastroparesis and may affect a patient's ability to tolerate oral intake. Even though no study has systematically assessed the impact of antiemetic therapy, 25% to 55% of patients with gastroparesis receive various antiemetics alone or in combination [6, 8, 10, 161]. A wide spectrum of different agents is available that can be given orally, rectally, transdermally or in injectable form (Table 3). All of these agents act through central pathways by targeting histamine 1, muscarinic, dopamine, neurokinin 1, or cannabinoid receptors within the brain, which explains their side effect profiles. In the absence of published data, the choice will depend on side effect profile, comorbid conditions, and symptom severity.

Pain is another challenging problem that often does not respond to dietary interventions or the use of prokinetics. Despite concerns about the use of opioids in benign disorders and their impact of gastric function and nausea, 25% to 40% of patients with gastroparesis are receiving opioids [6, 10, 14, 17, 49]. While a small case series suggested symptomatic improvement with venting gastrostomy [174], endoscopic or surgical tube placement carries a significant complication rate in these patients and should thus be reserved to patients who exhausted other options [57]. 

Antidepressants are commonly used in chronic pain syndromes and functional disorders of the gastrointestinal tract [175, 176]. Based on the symptomatic overlap between functional dyspepsia and gastroparesis, several reviews suggest the use of such agents, mostly tricyclic antidepressants, to improve nausea or pain [177–180]. The high prevalence of anxiety and/or depression and the importance of affect as a determinant of symptom severity provide an additional rationale for these centrally acting agents. However, case series show that more than one third of patients with gastroparesis receive at least one antidepressant [6, 8, 10], leaving fewer options to modify their medical therapy. Several agents have been tested in healthy volunteers with mixed results on postprandial symptoms [181–183]. A small retrospective study suggested significant improvement in about half of the patients [184]. However, more than 40% of these patients likely suffered from cyclical vomiting syndrome, a disorder that is clearly distinct from functional dyspepsia and gastroparesis. Two smaller trials compared amitriptyline with placebo in patients with functional dyspepsia and reported improvement of overall symptoms, but no change in pain, providing at least some support for this or related agents [27, 185]. Considering its appetite-stimulating and slight antiemetic effects, mirtazapine has been used in patients with gastroparesis [161, 186, 187]. A well-designed trial did not show superiority of venlafaxine over placebo in patients with functional dyspepsia [188]. Interestingly, the selective serotonin reuptake inhibitor fluoxetine improved symptoms only in depressed patients with functional dyspepsia, suggesting that global improvement with antidepressant therapy may be mediated through the beneficial effects on emotion and thus restricted to patients with affective spectrum disorders as recently also demonstrated in patients with irritable bowel syndrome [189, 190] (see Box 7).

### 7.6. Surgery and Gastroparesis

Considering the onset of gastroparesis after gastric surgery or vagotomy, operative intervention targeted a complete removal of the stomach in patients with postsurgical gastroparesis. While some retrospective case series reported subjective improvement in 70–80% of patients, up to one third of the patients died within 5 years after surgery [191, 192]. A small case series suggested some benefit in four of seven diabetic patients with refractory gastroparesis [193]. A more detailed analysis confirmed high complication rates and persisting symptoms in more than half of the patients, suggesting a limited utility of such radical approaches [194]. Using a rationale similar to the intrapyloric injection of botulinum toxin, surgeons have performed pyloroplasties to improve emptying. While short-term studies suggest a benefit [195], the results are similar to those of uncontrolled studies of botulinum toxin injection, which was shown to be ineffective in appropriately designed trials [76, 160]. Interestingly, patients with gastroparesis have an increased risk of undergoing abdominal surgeries [6], even though operative interventions are associated with a significant risk of complications and have no proven role in the management of this disorder.

### 7.7. Gastric Electrical Stimulation and Gastroparesis

The associations between changes in electrical activity of gastric muscle, gastric emptying and symptoms led to the development of electrical stimulation paradigms with the goal to entrain the rhythmic activity of the stomach, thereby normalizing gastric function. Initial experiments in animal models showed the feasibility of gastric electrical stimulation [196]. Using a frequency that was about 10% higher than the intrinsic frequency of gastric slow waves, gastric electrical activity could be successfully entrained in patients with initial results suggesting symptomatic improvement [197, 198]. However, entrainment of gastric slow waves did not correlate with changes in gastric emptying [199]. High frequency stimulation that did not aim at changing the intrinsic electrical activity of the stomach significantly increased contractile activity in a canine model and prompted the switch to currently used paradigm [200, 201]. An international open label study reported significant improvement of nausea in the majority of patients [202]. Several case series described similar findings with a decrease in hospitalization, less need for nutritional support and improved diabetic control in patients with diabetic gastroparesis [55, 203–207]. As gastric emptying is not significantly changed with gastric electrical stimulation [208], central mechanisms were proposed and presumably act though modulation of visceral sensory mechanisms in the brain. While preliminary studies indicate some changes in the activity of thalamus and caudate nucleus [209], gastric electrical stimulation does not affect brain activation during acute visceral stimulation [210].

Three randomized controlled trials have examined the effects of gastric electrical stimulation and have been fully reported. One study with temporary mucosal electrodes used a cross-over design [77], while the other two studies randomly assigned patients to periods with permanently implanted stimulators turned on or off, followed by an open label treatment phase [73, 211]. The temporary use of transmucosal stimulation did not show differences between the sham intervention and stimulation. The initial study of permanently implanted devices with serosal electrodes allocated patients to the two different treatment arms immediately after surgery and demonstrated a slight improvement of vomiting scores, primarily due to fewer emesis episodes of diabetic patients [73]. As the open label phase showed an ongoing improvement in all groups, the second study focused on diabetic patients and allowed patients to recover after surgery before randomly keeping the stimulator on or off. Unlike observed previously, there was a rapid improvement early after surgery without the additional benefit over time or differences between the two groups in the blinded phase of the trial [211].

Considering the available information many years after the initial introduction of gastric electrical stimulation, we are still lacking the definitive proof that the intervention is effective. The initial target has moved away from the stomach with the effects presumably due to modulation of central sensory processing in the brain. Despite this shift in proposed mechanisms, the electrodes are still implanted on the stomach, causing lead perforations and bowel obstructions in an admittedly small, but relevant number of patients [212, 213]. Reoperation rates of about 10% due to device-related complications reported from groups with extensive experience will likely underestimate the true complication rate once the approach is adopted more widely [55, 211]. The published data led some investigators to voice optimism [204, 211, 214, 215], whereas others emphasized the need for caution and more insight [216, 217]. Beyond better evidence of efficacy, we may need to rethink the approach. If neuromodulation of vagal afferent pathways indeed is the mechanism of action, then we may be able to move from *gastric* to *vagal* stimulation. With the course of the vagus and its branches, one could consider noninvasive approaches or use an access away from the abdominal cavity, thereby allowing a risk-free or at least lower risk approach (see Box 8).

## 8. Conclusion

Gastroparesis remains a relatively uncommon disease, mostly affecting women and significantly impairing their quality of life. The disorder is defined by chronic dyspeptic symptoms and delayed gastric emptying, which differentiates it from functional dyspepsia. Considering the importance of altered gastric motility as the endophenotype, the diagnosis requires an assessment of gastric emptying, typically in the form of a scintigraphic gastric emptying study for solid food. Despite extensive investigations, the majority of patients will suffer from gastroparesis without identifiable cause (idiopathic gastroparesis). A small subgroup may have had a preceding infection, pointing at the role of immune-mediated mechanisms in the pathogenesis of their illness. Recent mechanistic studies showed abnormalities in interstitial cells of Cajal or gastric innervation, which may open up additional venues to restore gastric function though stem cell therapy or use of growth factors. Despite the defining changes in motor function, delayed transit is not sufficient to explain the complex symptoms and should not be the sole target of therapeutic interventions. Combining dietary modifications with a selective use of prokinetics, symptom-oriented therapy and strategies that influence emotion as significant confounders may improve quality of life and avoid nutritional deficiencies in the majority of patients. The introduction of gastric electrical stimulation added a surgical option to our armamentarium. While open-label trials are encouraging, controlled investigations suggest a marginal benefit at best. As gastric electrical stimulation seems to affect symptoms through modulation of central processing rather than by altering gastric function, alternative and less invasive approaches are needed to directly target these mechanisms.

## Figures and Tables

**Box 1:**
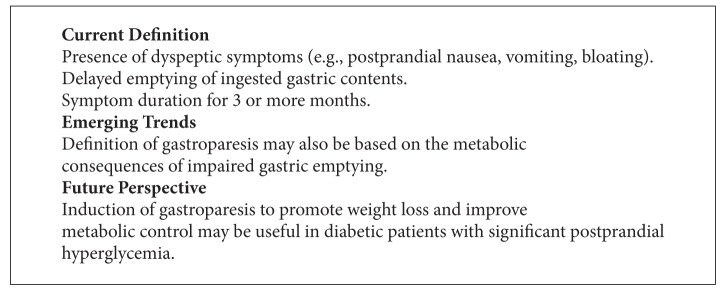


**Box 2:**
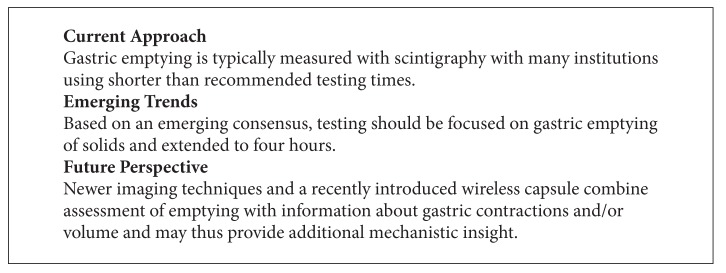


**Box 3:**
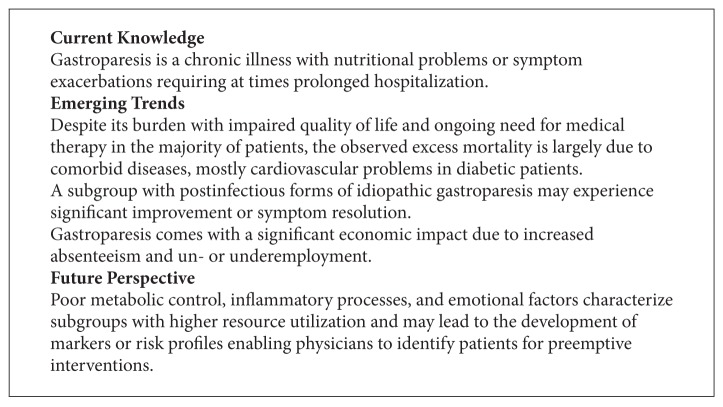


**Box 4:**
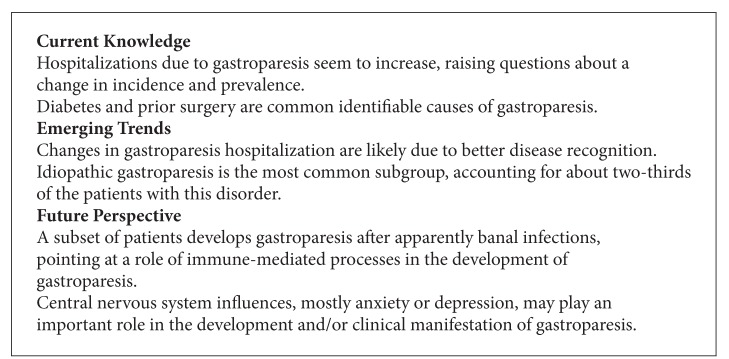


**Box 5:**
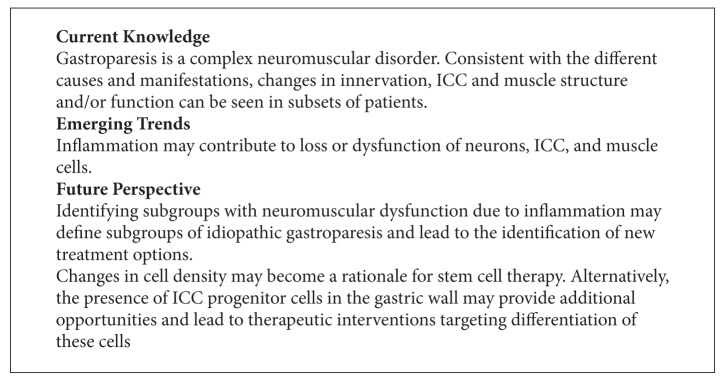


**Box 6:**
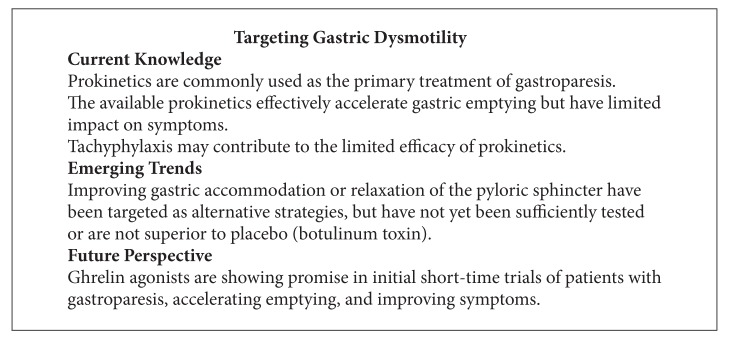


**Box 7:**
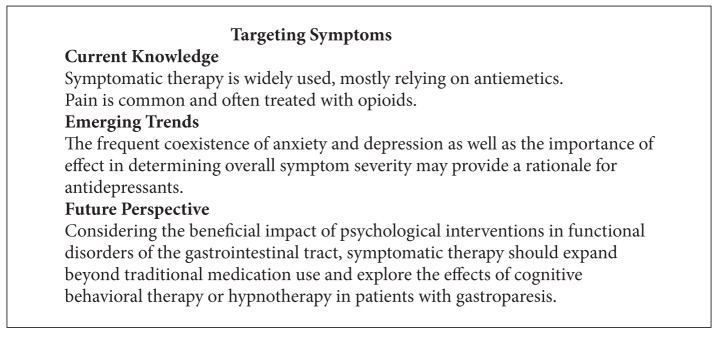


**Box 8:**
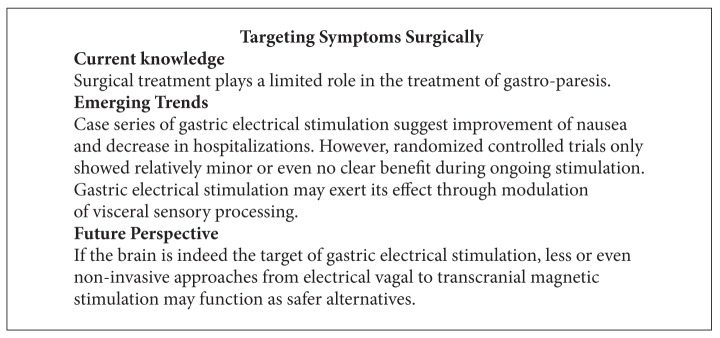


**Table 1:** Comparison of symptom severity scores obtained with a standardized questionnaire (PAGI-SYM) in patients with gastroparesis and functional dyspepsia [26–31].

Symptom	Functional dyspepsia	Gastroparesis
Pain: upper abdomen	2.9	3.5
	2.1	3.2
	2.5	2.3
	2.3	1.4
Pain: lower abdomen	1.3	1.6
	1.3	2.0
	1.5	1.7
	2.2	1.8
Nausea	2.6	3.4
	2.7	3.5
	1.3	2.3
	1.1	1.9
	3.5	3.3
Fullness	2.7	3.6
	3.0	3.9
	2.8	2.8
	2.1	2.5
	3.6	3.5
Bloating	2.8	3.3
	2.5	3.6
	2.7	2.6
	3.0	2.7
	3.3	3.1
Pyrosis	1.2	2.4
	1.8	2.3
	1.3	1.6
	1.3	1.7

**Table 2:** Distribution of different etiologies for gastroparesis in published case series.

Diabetic GP	Postsurgical GP	Idiopathic GP	Other	Sample (*n*)	Women	Year	Reference
39%	13%	36%		146	82%	1998	[15]
32%	17%	51%		28	60%	1999	[16]
52%		48%		33	75.8%	2003*	[73]
41%	3%	56%		63	84.1%	2005	[74]
50%		50%		106	79.3%	2007	[75]
56%	3%	41%		32	81.2%	2008	[76]
45%	10%	43%	2%	179	73.7%	2009	[50]
31%	8%	43%	18%	127	76.4%	2009	[54]
43%	8%	39%		63	78%	2009^#^	[63]
20%		53%	27%	55	80%	2009	[10]
26%		74%		68	85%	2010	[17]
33%		67%		299	81.9%	2010	[7]
64%	14%	22%		221	74%	2011*	[55]
25%	10%	61%	4%	326	80%	2011	[6]
22%	12%	62%		58	81.1%	2011*	[77]

*denotes studies of gastric electrical stimulation.

^#^denotes a study of hospitalized patients.

**Table 3:** Commonly used antiemetics agents with targets and potential adverse effects.

Agent	Target	Comment side effects	Comments	Reference
Scopolamine	M_1_ receptor	Visual disturbances dry mouth	Cognitive impairment in the elderly	[162, 163]
		Drowsiness		
		Urinary retention		
		Constipation		

Promethazine	H_1_ receptor	Sedation dystonia	Phenothiazine	[164]


Prochlorperazine	D_2_ receptor	Sedation dystonia	Phenothiazine Risk factor: dementia Tardive dyskinesia	[165, 166]
		Extrapyramidal motor dysfunction		
		Long QT syndrome		

Trimethobenzamide	D_2_ receptor	Sedation	Phenothiazine	[165, 166]
		Dystonia		
		Extrapyramidal motor dysfunction		

Metoclopramide	D_2_ receptor	Sedation	Tardive dyskinesia	[120, 126, 128, 130]
		Extrapyramidal motor dysfunction		
		Anxiety		

Ondansetron and granisetron	5-HT_3_ receptor	Headaches	Rare: QT prolongation	[167, 168]
		Constipation		

Aprepitant	NK_1_ receptor	Constipation Fatigue		[169, 170]


Dronabinol and nabilone	C_1/2_ receptor	Hypotension	Possible development of dependence	[171–173]
		Somnolence		
		Dysphoria	Cannabinoid-induced hyperemesis	
		Psychosis		

M_1_ receptor: acetylcholine M_1_ receptor antagonist; H_1_ receptor: histamine H_1_ receptor antagonist; D_2_ receptor: dopamine D_2_ receptor antagonist; 5-HT_3_ receptor: serotonin 5-HT_3_ receptor antagonist; NK_1_ receptor: neurokinin NK_1_ receptor antagonist; C_1/2_ receptor: cannabinoid C_1/2_ receptor agonist.
